# The Vitamin D Receptor Regulates Glycerolipid and Phospholipid Metabolism in Human Hepatocytes

**DOI:** 10.3390/biom10030493

**Published:** 2020-03-24

**Authors:** Teresa Martínez-Sena, Polina Soluyanova, Carla Guzmán, José Manuel Valdivielso, José Vicente Castell, Ramiro Jover

**Affiliations:** 1 Experimental Hepatology Unit, IIS Hospital La Fe, 46026 Valencia, Spain; 2 Vascular and Renal Translational Research Group, Experimental Medicine Department, IRBLleida, 25196 Lleida, Spain; 3 Departamento de Bioquímica y Biología Molecular, Facultad de Medicina, Universidad de Valencia, 46010 Valencia, Spain; 4 Centro de Investigación Biomédica en Red de Enfermedades Hepáticas y Digestivas (CIBERehd), ISCIII, 28029 Madrid, Spain

**Keywords:** human hepatocytes, lipid metabolism, vitamin D, vitamin D receptor

## Abstract

The vitamin D receptor (VDR) must be relevant to liver lipid metabolism because *VDR* deficient mice are protected from hepatosteatosis. Therefore, our objective was to define the role of VDR on the overall lipid metabolism in human hepatocytes. We developed an adenoviral vector for human VDR and performed transcriptomic and metabolomic analyses of cultured human hepatocytes upon VDR activation by vitamin D (VitD). Twenty percent of the VDR responsive genes were related to lipid metabolism, including *MOGAT1*, *LPGAT1*, *AGPAT2*, and *DGAT1* (glycerolipid metabolism); *CDS1*, *PCTP*, and *MAT1A* (phospholipid metabolism); and *FATP2*, *SLC6A12*, and *AQP3* (uptake of fatty acids, betaine, and glycerol, respectively). They were rapidly induced (4–6 h) upon VDR activation by 10 nM VitD or 100 µM lithocholic acid (LCA). Most of these genes were also upregulated by VDR/VitD in mouse livers in vivo. Ultra-performance liquid chromatography-tandem mass spectrometry (UPLC-MS) metabolomics demonstrated intracellular accumulation of triglycerides, with concomitant decreases in diglycerides and phosphatidates, at 8 and 24 h upon VDR activation. Significant alterations in phosphatidylcholines, increases in lyso-phosphatidylcholines and decreases in phosphatidylethanolamines and phosphatidylethanolamine plasmalogens were also observed. In conclusion, active VitD/VDR signaling in hepatocytes triggers an unanticipated coordinated gene response leading to triglyceride synthesis and to important perturbations in glycerolipids and phospholipids.

## 1. Introduction

Vitamin D (VitD) is a micronutrient but can also be synthesized in the skin using energy provided by UV-B radiation. Most of the biological effects of VitD are mediated by the VitD receptor (VDR, NR1I1), a member of the nuclear hormone receptor superfamily that forms a heterodimer with the retinoid X receptor and regulates target genes containing VDR response elements.

VDR expression has been identified in all major VitD target tissues, such as intestine, kidney, bone, and parathyroid gland [1], where it controls calcium and phosphorus homeostasis. However, about 3% of the mouse or human genome is regulated by the VitD endocrine system, suggesting a more widespread function for VDR [2].

The role of VDR in hepatocytes has not been completely defined yet, because hepatocytes express low levels of *VDR* mRNA, whilst non-parenchymal liver cells such as sinusoidal endothelial, Kupffer, and stellate cells do express higher levels [3]. Nevertheless, VDR mRNA and protein are expressed in human hepatocytes and HepG2 cells [4], where several typical hepatocyte genes are regulated by VitD via functional VDR response elements [5,6], reinforcing the notion that VDR activation and signaling in hepatocytes is also important. On top of that, hepatocyte VDR levels are induced by several conditions and stimuli: in human HepG2 cells, VDR expression is upregulated by free fatty acids, insulin, lithocholic acid (LCA), and VitD [4,7,8], and in human and mouse livers (e.g., apoE^-/-^ mice on high-fat diet (HFD) or mice on methionine-choline deficient diet) VDR is significantly induced in the settings of non-alcoholic fatty liver disease (NAFLD) [9].

Regarding the roles of VDR in hepatocytes, an association between VDR and bile acid metabolism has been demonstrated. LCA, a highly hydrophobic and toxic secondary bile acid, efficiently binds to VDR [10]. Activated VDR induces *CYP3A4* [5] and *SULT2A1* [6] to detoxify bile acids in the enterohepatic system. Moreover, the hepatocyte-specific *CYP7A1* gene, the rate-limiting enzyme in bile acid synthesis, is also controlled by VDR in human hepatocytes [4,11]. More recently, we have also proposed a new link between VDR and hepatic lipid metabolism as *VDR* deletion protected *apoE*^-/-^ mice from HFD-induced liver steatosis, which led us to postulate that hepatocyte VDR could play a key role in liver lipid metabolism and NAFLD pathogenesis [9]. In this context, we have also uncovered *ANGPTL8* as a novel VDR target gene that potentially contributes to higher triglyceride (TG) levels [8].

In the present study, we aimed to define the role of VDR on the overall lipid metabolism in hepatocytes. To this end, we developed an adenoviral vector for human VDR expression and performed whole transcriptomic and metabolomic analysis of human hepatocytes upon VDR activation. Our results uncover several novel VDR-target genes in human hepatocytes that coordinate an important perturbation in glycerolipid and phospholipid levels, which could have clinical relevance in prevalent diseases such as NAFLD.

## 2. Materials and Methods

### 2.1. Development of an Adenoviral Vector for Human VDR Expression

Ad-VDR was generated using the Adeno-X Adenoviral System 3 kit (Takara #632269, Conda Laboratories, Madrid, Spain), following the manufacturer’s instructions. The *VDR* cDNA was obtained by RT-PCR from HeLa cells total RNA using the primers:

5′-gtaactataacggtcCACCCCTGGGCTCCACTTACC-3′ (forward)
5′-attacctctttctccCCGCCACAGGCTGTCCTAGTC-3′ (reverse)

After obtaining the recombinant adenovirus in HEK293T cells, it was purified using the Vivapure AdenoPACK 20 kit (VS-AVPQ022; Sartorius, Madrid, Spain). Subsequently, the virus titer was determined by plaque-forming assay.

The functionality of the expressed VDR was assessed in human upcyte hepatocytes and HepG2 by measuring the expression of *CYP24A1*, a well-characterized VDR target gene. Results in Supplementary Figure S1 show that *CYP24A1* was highly induced when transfected VDR was activated. The large fold-increase observed is consequence of the very low basal mRNA level of *CYP24A1*, which is almost null in the absence of active VDR signaling.

### 2.2. Culture of Human Upcyte Hepatocytes and HepG2 Cells

Second generation upcyte hepatocytes and their specific culture mediums were obtained from upcyte Technologies GmbH (Hamburg, Germany) [12]. Hepatocytes were thawed in Thawing Medium and seeded at a density of 5000 cells/cm^2^ in collagen-type I-coated flasks (Corning, New York, NY, USA). Cells were cultured in Hepatocyte Culture Medium, until they reached 80% confluence. Medium was replaced every 2 days. Cells were subcultured with 0.25% trypsin/0.02% EDTA (Gibco BRL/Thermo Fisher Scientific, Madrid, Spain) and were seeded at a density of 70,000 cells/cm^2^ in collagen-type I-coated 12-well plates (Corning, New York, NY, USA) in Hepatocyte Culture Medium. After 4–5 h, the medium was replaced with Hepatocyte High Performance Medium.

HepG2 cells (ATCC, Rockville, MD, USA) were cultured in Ham’s F-12/Leibovitz L-15 (1:1, *v/v*) medium (Gibco BRL/Thermo Fisher Scientific, Madrid, Spain) supplemented with 7% newborn calf serum, 2 mmol/L L-glutamine, 50 U/mL penicillin, and 50 mg/mL streptomycin (Sigma Aldrich, Madrid, Spain).

For adenoviral infection, we selected upcyte hepatocytes from donors with a low level of endogenous VDR [13]. Similarly, HepG2 cells have a negligible VDR expression [13]. Cells were incubated with a non-cytotoxic dose of 1 plaque-forming unit/cell of adenoviral vectors: Ad-VDR, encoding human *VDR*, or Ad-C, an insertless adenovirus. This dose of Ad-VDR increases VDR expression to levels comparable to those found in NAFLD livers [8,9]. Forty-eight hours later, cells were shifted to adenovirus-free medium and cultured for an additional period of time in the presence of VDR agonists: VitD (1a,25-dihydroxyvitamin D3/calcitriol, Sigma Aldrich, Madrid, Spain) or LCA (lithocholic acid, Sigma Aldrich, Madrid, Spain). Stock solutions were prepared in DMSO (Sigma Aldrich, Madrid, Spain), and added to cultured cells at a final concentration of 10 nM and 100 µM, respectively, for a variable period of time as indicated.

### 2.3. Animals and Treatments

The *apoE*^−/−^ mouse on a high fat diet (HFD) containing cholesterol represents a fast model displaying all characteristic features of NAFDL and metabolic syndrome. The *apoE*^−/−^ and *apoE*^−/−^*Vdr*^−/−^ mice were generated as described elsewhere [9]. Mice of both sexes were used. After weaning at 21 days, *apoE*^−/−^ mice were maintained on a regular mouse chow (Harlan Teklad, Madison, WI, USA), while *apoE*^−/−^*Vdr*^−/−^ mice were fed a high-calcium rescue diet (20% Lactose, 2% Ca, 1.25% P; TD.96348, Harlan Teklad, Madison, WI, USA) to prevent hypocalcaemia. At 12 weeks of age, *apoE*^−/−^ and *apoE*^−/−^*Vdr*^−/−^ were placed on a HFD, containing 21% fat, 0.75% Cholesterol, 20% Lactose, 2% Ca, 1.25% P (S9358-E010, Ssniff Spezialdiäten GmbH, Soest, Germany) and drinking water with 1% Ca-gluconate, and were kept for additional 8 weeks. For paricalcitol supplementation, a subgroup of the *apoE*^−/−^ mice were injected intraperitoneally with 0.75 µg/kg of 19-nor-1,25-dihydroxyvitamin D2 (paricalcitol Zemplar, AbbVie, North Chicago, IL, USA) 3× weekly, for the 8 weeks on HFD.

Mice were euthanized at 20 weeks of age. The animals were perfused with PBS through the left ventricle and one part of the liver was snap-frozen.

All animal studies were approved by the local Animal Ethics Committee in accordance with the guidelines of European Research Council for the care and use of laboratory animals (n° EV204-2016).

### 2.4. Transcriptomic Analyses and Quantitative RT-PCR

Three independent HepG2 cultures were transfected with Ad-VDR for 48 h, and then were treated with 10 nM VitD for 4 h. Total cellular RNA was extracted with the RNeasy Plus minikit (Qiagen, Madrid, Spain), which removes contaminating genomic DNA. The integrity of purified RNA was estimated by microcapillary electrophoresis (2100 Bioanalyzer, Agilent Technologies, Santa Clara, CA, USA). RNA Integrity Numbers were all above 9.3.

Genome-wide expression profiling was performed using the human Clariom™S Assay (Affymetrix, Thermo Fisher Scientific, Madrid, Spain), which interrogates over 20,000 well-annotated genes. Genechip hybridization and scanning were performed at the Gene Analysis Service (Central Research Unit UCIM, Faculty of Medicine, University of Valencia).

The data was normalized using the RMA method, and probes were summarized using the revised entrez-based probe annotation [14]. A probe-filtering step excluding those probes that show a lower coefficient of variation or low intensity levels in all observations was employed. Differential expression analysis was assessed using linear models for microarray data [15] based on empirical Bayes moderated t-statistics for all filtered probe sets. To correct for multiple testing, the false discovery rate (FDR) was estimated from *p*-values derived from the moderated t-statistics using the method of Benjamini and Hochberg [16]. The microarray data have been deposited in NCBI’s Gene Expression Omnibus (GEO) and are accessible through the accession number GSE138376.

For RT-qPCR gene expression analyses, in HepG2, upcyte hepatocytes and mouse livers, total RNA (1 µg) was reverse transcribed using the Moloney murine leukemia virus reverse transcriptase (Invitrogen/Thermo Fisher Scientific, Madrid, Spain) following the manufacturer’s protocol. Diluted cDNA (3 µL) was amplified with a rapid thermal cycler (LightCycler Instrument LC480, Roche Diagnostics, Barcelona, Spain) in 15 µL of LightCycler DNA Master SYBR Green I (Roche Diagnostics, Barcelona, Spain) and 0.3 µM of each primer (Supplementary Table S1). In parallel, we always analyzed the mRNA concentration of the human or mouse housekeeping porphobilinogen deaminase (*PBGD*), glyceraldehyde 3-phosphate dehydrogenase (*GAPDH*) and ribosomal protein lateral stalk subunit P0 (*RPLP0*) as internal controls for normalization.

### 2.5. Ultra-Performance Liquid Chromatography-Tandem Mass Spectrometry (UPLC-MS) Analysis

Cultured upcyte hepatocytes (*n* = 4) were infected with Ad-VDR for 48h, and then 60 µM fatty acids (oleate:palmitate, 2:1) and 10 nM VitD were added for 8 or 24 h. Cells are washed and scraped in 600 μL of cold PBS containing 0.375 μg/mL of Reserpine as an internal standard. Next, homogenates were frozen, thawed and vortexed three times to increase cell disruption and metabolite extraction. Thereafter, they were extracted twice with 2 volumes of Methanol: Chloroform (1:2). The organic fraction was split into two aliquots that were evaporated under vacuum (SpeedVac SPD121, Thermo Fisher Scientific, Madrid, Spain) and re-dissolved in 75 μL of 1 μM internal standard solution (Phenylalanine-D_5_, Tryptophan-D_5_, and Caffeine-D_9_) matching the initial composition of the mobile phases used for metabolomic and lipidomic analysis. Quality control (QC) samples were a pool of all samples for each chromatographic mode.

Chromatographic analysis was performed on a 6550 iFunnel Q-TOF chromatograph (Agilent Technologies, Santa Clara, CA, USA) using a Kinetex C18 column (100 × 2.1 mm, 1.7 µm, Phenomenex, Torrance, CA, USA) coupled to an ACQUITY UPLC BEH C18 VanGuard Pre-column (5 × 2.1 mm, 1.7 µm, Waters, Milford, MA, USA). To increase coverage, two different procedures were followed: (**a**) Metabolomic Analysis, with a mobile phase A (H_2_O, 0.1% *v/v* HCOOH), a mobile phase B (CH_3_CN, 0.1% *v/v* HCOOH) and a gradient elution performed as follows: 0–0.5 min 2%B, 0.5–4 min 20%B, 4–9 min 95%B, 9–12 min 2%B; and (**b**) Lipidomic Reversed Phase Analysis, with a gradient elution performed as described elsewhere [17].

Full scan MS data from 70 to 1200 *m*/*z* was collected on an iFunnel quadrupole time of flight (QTOF) Agilent 6550 spectrometer (Agilent Technologies, Santa Clara, CA, USA). Electrospray ionization parameters, MS spectra calibration, MSMS analysis settings, identification of metabolites and data processing is extensively detailed in Supplemental UPLC-MS methods.

### 2.6. Statistics

Quantitative variables were expressed as mean ± standard error of the mean. The significance of the differences among groups was evaluated by one-way ANOVA followed by Tukey’s post hoc analysis. Significance was set at a value of *p* < 0.05.

## 3. Results

### 3.1. Transcriptomic Analysis of Human HepG2 Cells with Activated VDR

**Figure 1:**
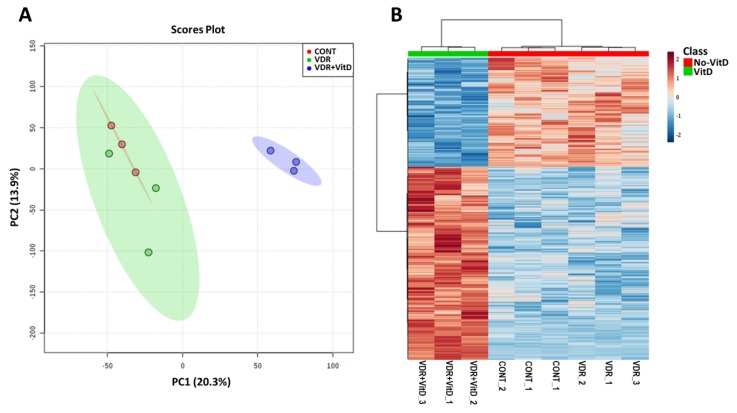
Genome-wide expression profiling of HepG2 cells with activated VDR. HepG2 cells were transfected with Ad-C or Ad-VDR for 48 h. Then, 10 nM Vitamin D (VitD) (VDR+VitD) or vehicle (VDR & CONT) were added for 4h. Total RNA was purified and expression profiling was performed by microarray analysis. Non-informative mRNAs in the dataset were filtered by IQR and 8000 mRNAs were left for further analysis. Mean-centred and SD autoscaling were performed. (**A**) Principal component analysis. (**B**) Hierarchical clustering showing the top 1000 differentially expressed genes.

We performed genome-wide expression profiling of HepG2 cells transfected with Ad-VDR and exposed to VitD for 4 h. Principal component analysis (PCA) and hierarchical clustering (Figure 1) revealed that increased expression of VDR by Ad-VDR did not cause a significant perturbation in the transcriptome as compared with cells transfected with a control adenovirus. However, VitD stimulation of Ad-VDR in HepG2 cells triggered an extensive alteration in the cell expression profile.

Comparative analysis (VitD vs. no VitD) demonstrated that 298 genes were significantly altered by activated VDR (fold-change > 1.5 or < 1.5, FDR-adjusted *p-*value < 0.05). The main effect of VitD-activated VDR was gene upregulation as 229 genes were induced, whereas only 69 genes were repressed.

Analysis of the 298 VitD-regulated genes in terms of functional annotation and gene ontology enrichment (ConsensusPathDB [18]) revealed 106 enriched gene ontology-based sets (over-representation in levels 4 and 5 of the category of Biological Processes, *p* < 0.005, q < 0.05) from which 21 (20%) were related to lipid metabolism (Table 1).

Therefore, results indicate that VitD causes a significant transcriptomic alteration in VDR-expressing HepG2 cells, and a substantial number of the altered genes (20%) are related to lipid metabolism.

### 3.2. Novel Genes Regulated by VDR in Human Hepatocytes

New potential VDR target genes were selected from the list of differentially expressed genes. We focused on 10 of them, which are involved in lipid metabolism and have not been previously associated with VitD: *MOGAT1*, *DGAT1*, and *AGPAT2* (associated with glycerolipid metabolism and TG synthesis), *LPGAT1*, *CDS1*, *PCTP*, and *MAT1A* (involved in phospholipid metabolism), and *FATP2*, *SLC6A12*, and *AQP3* (responsible for the uptake of fatty acids, betaine and glycerol, respectively) (see Supplementary Table S2 for official gene names and roles).

**Figure 2:**
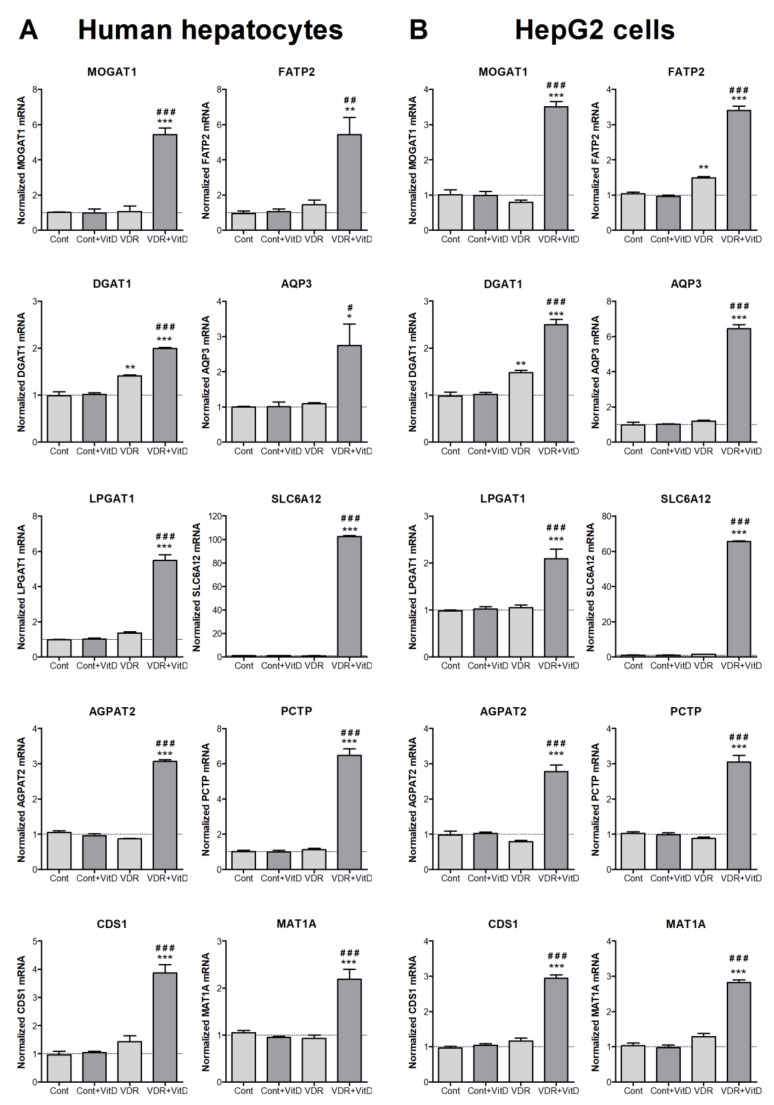
New VitD-responsive genes related to lipid metabolism in human hepatic cells. Cultured upcyte hepatocytes (**A**) or HepG2 cells (**B**) were infected with Ad-VDR (VDR—VitD receptor) or with a control adenovirus (Cont) and, 48 h later, 10 nM VitD was incorporated for 4 h. mRNA levels were determined by RT-qPCR and normalized with the housekeeping porphobilinogen deaminase (*PBGD*) and ribosomal protein lateral stalk subunit P0 (*RPLP0*) mRNAs. Data represent the mean ± SEM relative to cells transfected with the control adenovirus (Cont) from 3–4 independent experiments. *p < 0.05, **p < 0.01 and ***p < 0.001 VDR vs. no VDR; #*p* < 0.05, ##*p* < 0.01 and ###*p* < 0.001 VitD vs. no VitD.

Experimental validation by RT-qPCR was performed in both HepG2 cells and human upcyte hepatocytes. Cells were first transduced with Ad-VDR and, 48 h later, they were treated with VitD for 4 h. Results in Figure 2 confirmed the VDR-dependent upregulations observed by microarray expression analysis in HepG2. Moreover, results demonstrated that the effects observed in HepG2 cells are accurately reproduced in cultured human upcyte hepatocytes.

Other well characterized agonist for VDR is LCA, a secondary hydrophobic bile acid. We tested next if this enterohepatic VDR agonist was able to induce the novel VitD-responsive genes in HepG2 cells. Results in Supplementary Figure S2 show the dose-response curves of these 10 genes to LCA. All them were activated and, for most of them, 100 µM LCA was the concentration causing the highest induction.

**Figure 3:**
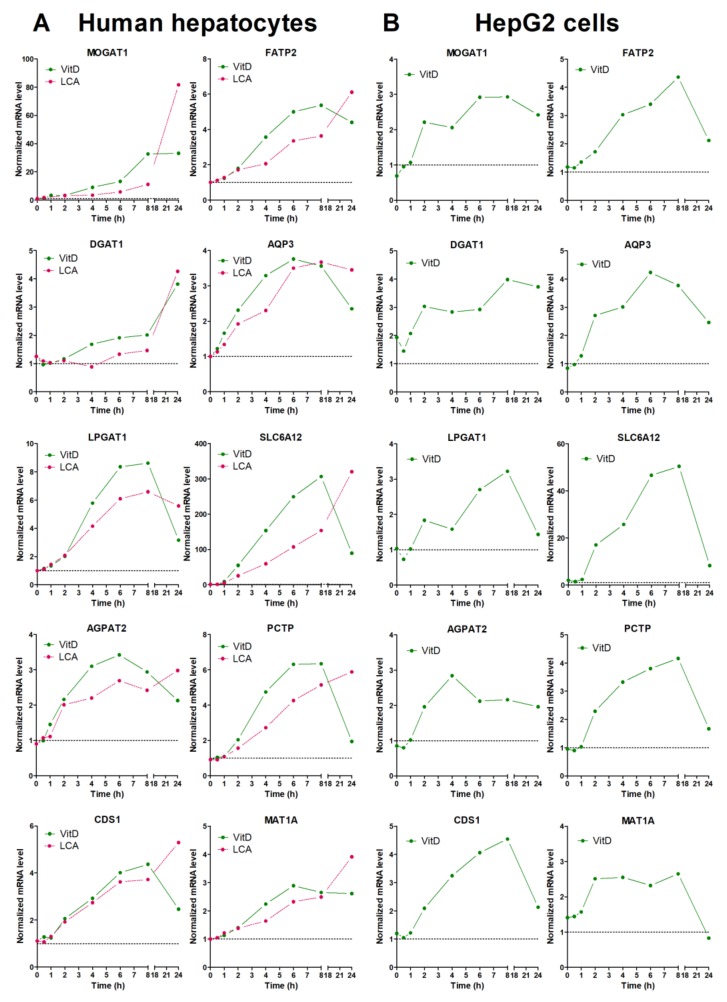
Time-course response to VitD and LCA of novel VDR-responsive genes. Cultured upcyte hepatocytes (**A**) or HepG2 cells (**B**) were infected with Ad-VDR and, 48 h later, 10 nM VitD or 100 µM LCA was incorporated for different times. mRNA levels were determined by RT-qPCR and normalized with the housekeeping *PBGD* & *RPLP0* mRNAs. Data represent the expression level relative to cells transfected with the control adenovirus (dotted line = 1).

Finally, we performed time-course analysis of VDR-response genes in both upcyte hepatocytes and HepG2 (Figure 3). After VitD, most of the genes showed a time-course expression profile that was quite similar in both cell models. The most remarkable difference was seen in *MOGAT1* that showed a much more prominent induction in upcyte hepatocytes. Most of the genes reached maximal expression levels between 6 and 8 h after VitD, and underwent a clear decrease by 24 h. This decrease could be due to the parallel induction of CYP24A1 that inactivates VitD (see Supplementary Figure S1). One exception was *DGAT1*, which reached maximal levels of expression at 24 h after VitD in upcyte hepatocytes (Figure 3).

The response of LCA was similar to that of VitD at early time points, but the LCA-dependent upregulation continued until 24 h, where LCA caused maximal effects (Figure 3). The different response to VitD and LCA at 24 h could be explained by the persistence (lack of clearance) of LCA in the culture medium.

### 3.3. Expression and Regulation of Novel VDR-Responsive Genes in Mouse Liver

To investigate the potential regulation of these novel genes by VDR in vivo, we performed mRNA expression analysis in the livers of three different groups of mice: VDR-deficient, control, and VitD (paricalcitol) supplemented mice, all them on an *apoE*^−/−^ background and on a HFD for 8-weeks. At the end of the HFD, the liver TG levels were 220 ± 68, 446 ± 71 and 510 ± 91 μg TG/mg protein (mean ± SD) in *apoE* & *Vdr* double knock-out, *apoE*^−/−^, and *apoE*^−/−^ + VitD, respectively.

**Figure 4:**
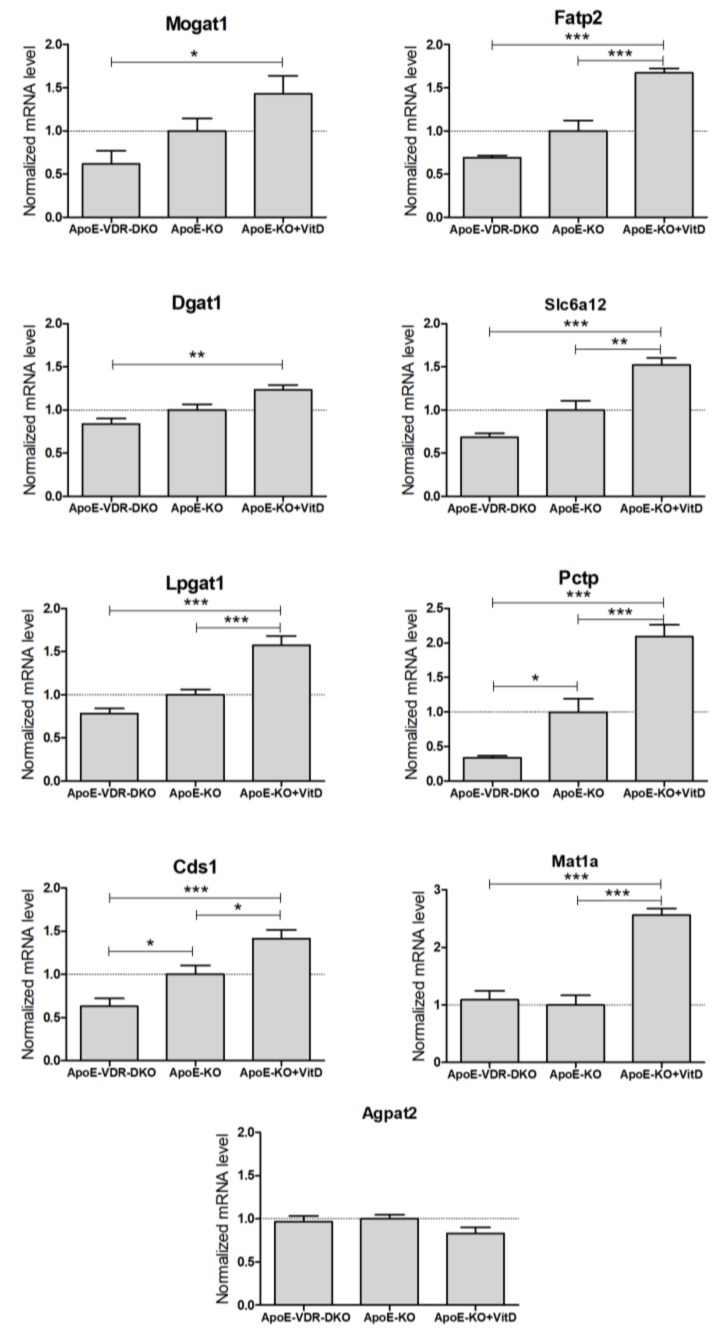
Expression of novel VDR-regulated genes in the livers of VDR-deficient and VitD-treated mice. Twelve-week-old mice from three groups: *apoE*^−/−^ (ApoE-KO, *n* = 18), *apoE* & *Vdr* double knock-out (ApoE-VDR-DKO, *n* = 10) and *apoE*^−/−^ treated with paricalcitol (ApoE-KO+VitD, *n* = 8), were placed on a HFD for 8 weeks. Total liver RNA was isolated, and the mRNA levels were determined by RT-qPCR and normalized with the housekeeping *Gapdh* & *Rplp0* mRNAs. Data represent the mRNA level as mean ± SEM relative to *apoE*-KO mice (dotted line = 1). *p < 0.05, **p < 0.01 and ***p < 0.001.

We were unable to amplify *Aqp3* from mouse liver, which is in agreement with the low expression reported in mouse liver [19] and with the substantial expression of *AQP3* reported in human liver [20,21]. Results demonstrate that 8 of the 9 detected mouse genes had significant differences in hepatic expression between VDR-deficient and VitD-supplemented mice (Figure 4). *Agpat2* was the only mouse liver gene unresponsive to VDR expression and VitD. All the other detected genes showed higher expression in the VitD-supplemented mice, supporting the notion that they could be novel positive VDR target genes in vivo (Figure 4).

*ANGPTL8*, another VDR target gene involved in TG homeostasis [8], was repressed by 70% in the livers of *apoE* & *Vdr* double knock-out mice vs. *apoE*^−/−^ mice (data not shown).

### 3.4. Metabolomic Alterations Caused by Activated VDR in Human Hepatocytes

Human upcyte hepatocytes were transfected with Ad-VDR and exposed to 10 nM VitD, in the presence of 60 µM oleate:palmitate (2:1), for 8 and 24 h. Cell extracts were separated by two different chromatographic methods followed by UPLC-MS analysis. A total of 1309 features were recorded and 272 + 218 signals were identified from the two methods.

Unsupervised multivariate analysis of the dataset was performed. The two PCAs, in Supplementary Figure S3, demonstrate two main sources of variation, one associated with the effect of time (8 and 24h, Supplementary Figure S3A) and other with the effect of VitD (VDR and VDR+VitD, Supplementary Figure S3B).

**Figure 5:**
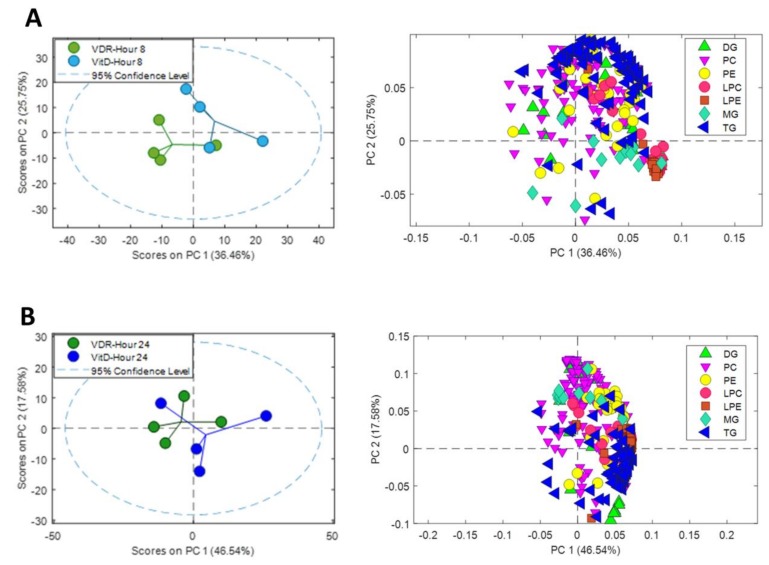
Metabolomic PCA scores plots of human upcyte hepatocytes with VitD-activated VDR. Left: Scores of VDR and VDR+VitD (VitD) hepatocytes after 8 (**A**) and 24 (**B**) h incubation. Right: Loadings for identified triglycerides (TG), diacylglycerols (DG), monoacylglycerols (MG), phosphatidylcholines (PC), phosphatidylethanlolamines (PE), lysophosphatidylcholines (LPC) and lysophosphatidylethanlolamines (LPE) after 8h (**A**) and 24h (**B**).

In order to facilitate the visualization and to simplify the interpretation of the data, we performed separate PCAs at 8 and 24 h after VitD, including only the signals of identified metabolites from the major lipid classes (TG, diacylglycerols (DG), monoacylglycerols (MG), phosphatidylcholines (PC), phosphatidylethanlolamines (PE), lysophosphatidylcholines (LPC) and lysophosphatidylethanlolamines (LPE)) (Figure 5). A source of variation was observed between VDR cells and VDR cells treated with VitD both after 8 (Figure 5A) and 24h (Figure 5B) incubation.

Analysis of the loadings of the identified lipid metabolites demonstrated the contribution of the different lipid classes to the separation between the two conditions: VDR alone vs. VDR+VitD. More specifically, intracellular TGs showed a substantial influence, both at 8 and 24h. The levels of many lysophospholipids (primarily LPC) have also a significant contribution at 8h after VitD. Moreover, differences in the levels of phospholipids seem to have an important influence in group discrimination (Figure 5).

**Figure 6:**
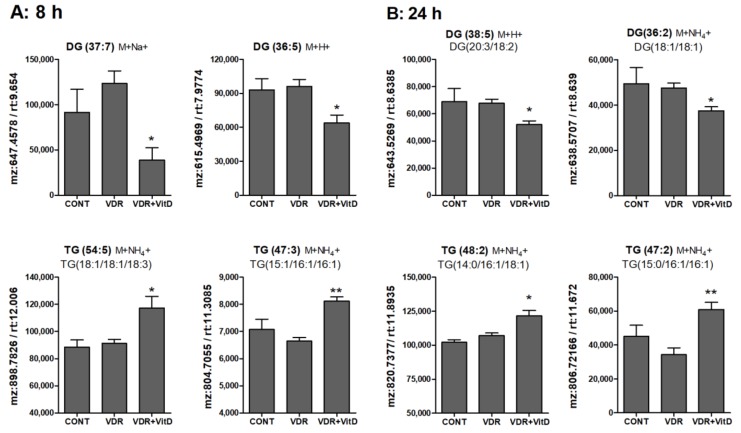
Relative intracellular levels of selected TGs and DGs in cultured human hepatocytes exposed to VitD. Upcyte hepatocytes were transfected with an insertless adenovirus Ad-C or with Ad-VDR for 48 h. Then, 10 nM VitD (VDR+VitD, *n* = 4) or vehicle (CONT, *n* = 3 and VDR, *n* = 4) were added for 8 (**A**) or 24 h (**B**). Cells were washed, and intracellular metabolites extracted and analyzed by ultra-performance liquid chromatography-tandem mass spectrometry (UPLC-MS) as described in Materials and Methods. Data represent the normalized chromatographic peak areas of each feature (characterized by a mass-to-charge ratio (mz) and a retention time (rt)) and are expressed as mean ± SEM. *p < 0.05 and **p < 0.01 VDR+VitD vs. VDR.

**Figure 7:**
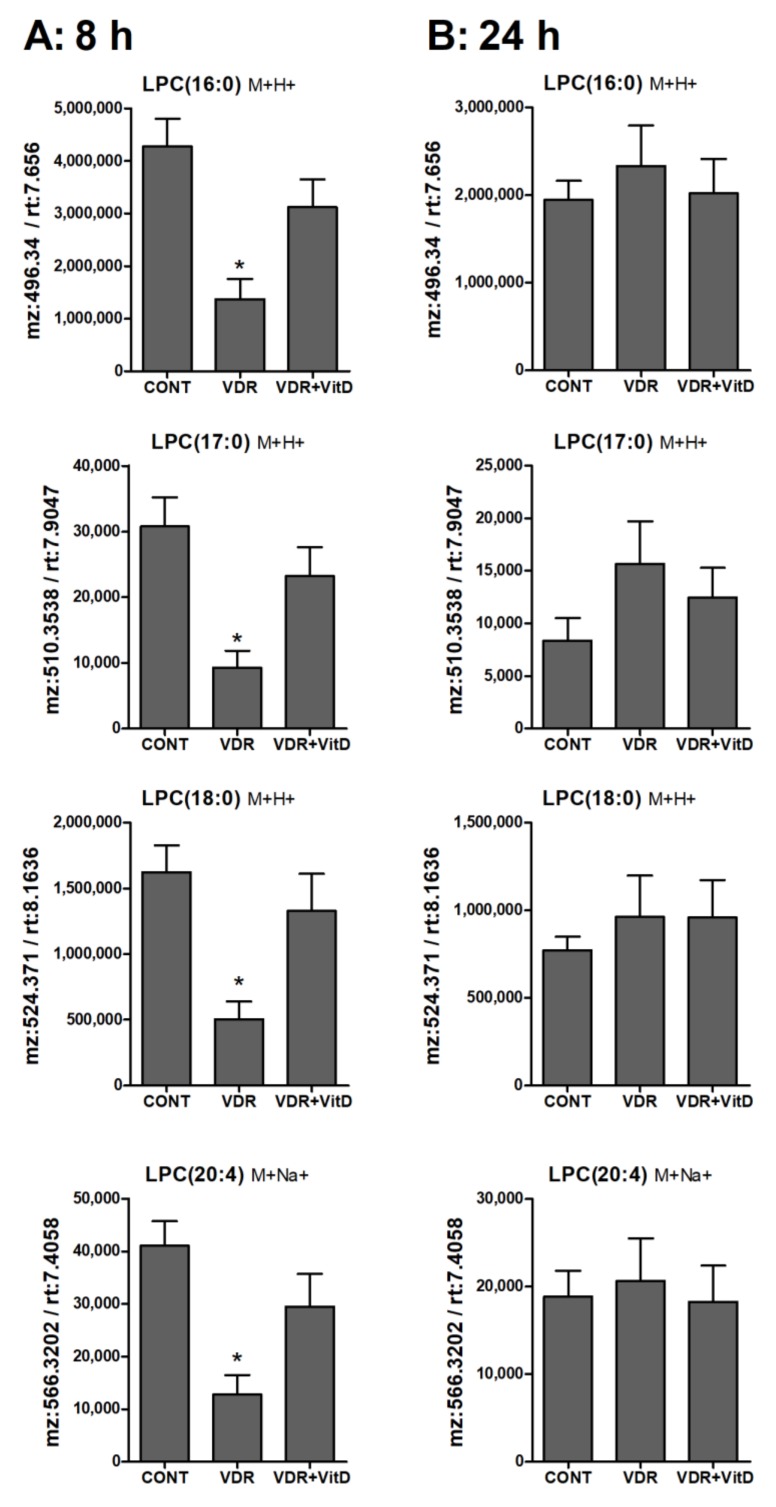
Relative intracellular levs of selected lysophosphatidylcholines in cultured human hepatocytes exposed to VitD. Upcyte hepatocytes were transfected with control Ad-C or with Ad-VDR for 48 h. Then, 10 nM VitD (VDR+VitD, *n* = 4) or vehicle (CONT, *n* = 3 and VDR, *n* = 4) were added for 8 (**A**) or 24 h (**B**). Cells were washed, and intracellular metabolites extracted and analyzed by UPLC-MS as described in Materials and Methods. *p < 0.05 VDR vs. VDR+VitD.

**Figure 8:**
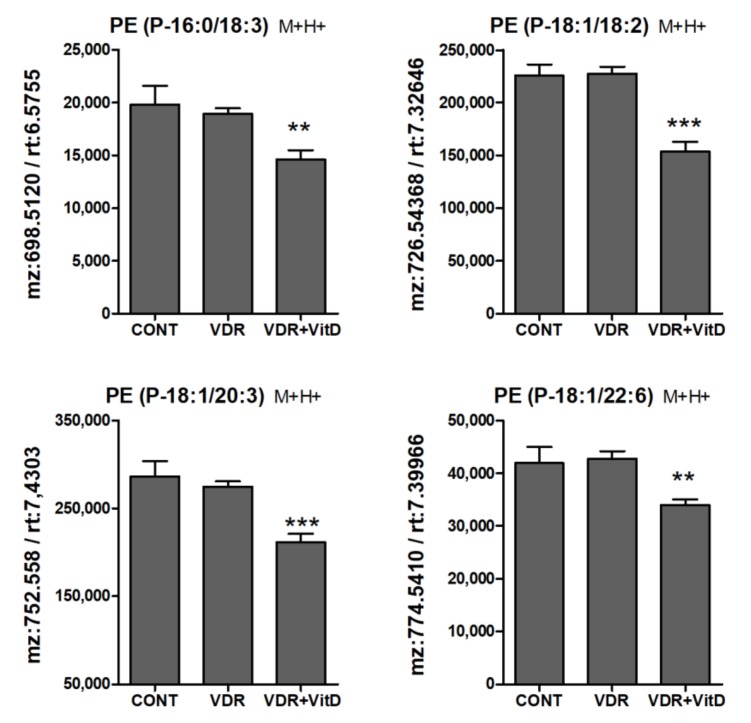
Relative intracellular levs of PE alkenyl (plasmenyl) ethers in cultured human hepatocytes exposed to VitD. Upcyte hepatocytes were transfected with a control Ad-C or with Ad-VDR for 48 h. Then, 10 nM VitD (VDR+VitD, *n* = 4) or vehicle (CONT, *n* = 3 and VDR, *n* = 4) were added for 24 h. Cells were washed, and intracellular metabolites extracted and analyzed by UPLC-MS as described in Materials and Methods. *p < 0.05, **p < 0.01 and ***p < 0.001 VDR+VitD vs. VDR.

Results in Figure 6, Figure 7 and Figure 8 show the alteration of specific lipid species after VitD.

In general terms, DGs were decreased whereas TGs were increased by VitD at 8 (Figure 6A) and 24 h (Figure 6B). Several MGs species were also identified and, similarly to DGs, they also consistently decreased after VitD, however the variability among replicates was large and no statistical significance was found (data not shown).

Regarding phospholipids, many PC species appeared significantly altered in hepatocytes treated with VitD, both at 8 and 24 h. Some of them were increased but others were decreased. Therefore, VitD may induce a remodeling of the PC pool. Similar results were observed for PEs, but in this case we only found statistically significant differences in decreased PE species after VitD (Supplementary Figure S4).

Lysophospholipids, and particularly LPC, showed higher levels in 8 h VitD-treated hepatocytes (Figure 7A), but, after 24 h, differences between these two conditions were no longer evident (Figure 7B).

Another lipid class that was significantly altered by VitD was the PE plasmalogens (PE alkenyl ethers). They were consistently decreased upon 10 nM VitD incubation, particularly after 24 h (Figure 8).

Finally, regarding other metabolites it is worth mentioning that hepatocytes transfected with VDR and exposed to VitD demonstrated higher levels of betaine and lower levels of several phosphatidic acid (PA) species (Supplementary Figure S5).

In summary, VitD triggers the activation of genes involved in the uptake of fatty acids, glycerol, and betaine, and in the metabolism of glycerolipids and phospholipids. The activation of this gene network results in decreased levels of PA, DG, MG, PE, and PE plasmalogens, and in increased levels of betaine, LPC, and TG. Moreover, many PC species decreased whereas other increased in a likely remodeling of the PC pool.

## 4. Discussion

We have previously shown, in a mouse model, a causal relationship between hepatocyte VDR and liver TG [9]. We also searched for gene candidates involved in this VDR-dependent effect and found some lipid metabolism genes (e.g., *DGAT2* or *CEBPA*) that responded to plasmid-transfected VDR in HepG2 [9]. Another uncovered VDR-target gene involved in lipid metabolism was *ANGPTL8* [8]. In the present study, we performed whole transcriptome analysis after VDR activation in human hepatocytes to identify the whole repertoire of potential VDR-dependent lipid metabolism genes. The main effect of VitD-activated VDR was the upregulation of more than 200 genes, from which 20% were related to lipid metabolism. We have focused on ten of them with the more significant response.

TGs are synthesized by two major pathways, the glycerol 3-phosphate pathway and the MG pathway [22]. Both pathways generate DG. In the glycerol 3-phosphate pathway DG is synthesized by dephosphorylation of PA, whereas in the MG pathway, DG is formed from MG and fatty acyl-CoA by MOGATs. The new synthesized DG can be used as a substrate for TG synthesis by DGATs [22,23].

Our results demonstrate that VDR induces the expression of *AGPAT2* (glycerol 3-phosphate pathway) as well as *MOGAT1* and *LPGAT1* (MG pathway). Moreover, *DGAT1* (this study) and *DGAT2* [9] are also upregulated, which suggest a coordinated response directed to TG synthesis in hepatocytes.

AGPAT2 is a 1-acylglycerol-3-phosphate acyltransferase that acylates Lyso-PA at the sn-2 position with fatty acyl-CoAs to generate PA. AGPAT2 seems to be a key enzyme in the synthesis of TG via the glycerol 3-phosphate pathway as hepatic AGPAT activity is reduced 90% in *AGPAT2* knockout mice [24]. Our results demonstrate that *AGPAT2* is significantly induced in human hepatocytes and HepG2 cells after VDR activation. However, its expression was not altered in the livers of *Vdr*^−/−^ mice or after VitD supplementation, which suggest that the regulation of *AGPAT2* by VDR could be specific for the human gene.

The synthesis of TG via the MG pathway is also active in human liver, where three MOGAT genes are expressed [25]. A liver-specific shRNA against *Mogat1* protected the liver from steatosis in three different models [26]. This suggest that MOGAT1 could be an important determinant for TG accumulation in hepatocytes. Activated VDR not only induced *MOGAT1* but also *LPGAT1*, which is also an acyltransferase that belongs to the lyso-PA acyltransferase family [27]. LPGAT1 may be involved in the remodeling of lysophosphatidylglycerol back to phosphatidylglycerol. However, Hiramine et al. [28] demonstrated that LPGAT1 also displays MOGAT activity and hence contributes to the synthesis of DG and TG. Indeed, liver MOGAT activity was significantly reduced in *db*/*db* mice infected with *LPGAT1* shRNA adenovirus [28]. So, it is probable that LPGAT1 functions either as MOGAT or LPGAT enzyme depending on the physiopathological context. Our results demonstrate that activation of VDR in hepatocytes leads to the upregulation of two important genes for DG synthesis through the MG pathway: *MOGAT1* and *LPGAT1*.

DGAT1 and DGAT2, catalyze the final esterification of a fatty acyl moiety to a DG molecule to produce TG. These enzymes commit DG to being stored as TG rather than being channeled to the synthesis of phospholipids. Both DGAT enzymes may contribute to the overt activity responsible for the synthesis of TG for cytosolic lipid droplets, and both may also contribute, through different mechanisms, to the synthesis of TG for lipid droplets at the lumen of the endoplasmic reticulum and for the nascent very low-density lipoprotein (VLDL) precursor [29]. Both, *DGAT1* (this study) and *DGAT2* [9] were upregulated by activated VDR, which is in agreement with a recent study showing that calcitriol treatment increased mRNA expression of *DGAT1* and *DGAT2* in C2C12 myotubes [30]. The upregulation of *DGAT1/2* along with the concomitant induction of *AGPAT2*, *MOGAT1*, and *LPGAT1*, suggest that the activated VDR can trigger a coordinated response promoting the synthesis of TG in human hepatocytes.

Another novel VDR-response gene, *PCTP*, which is enriched in hepatocytes, exchanges PCs among membranes. PCTP might function within the hepatocyte to redistribute PCs from the endoplasmic reticulum to lipid droplets thus regulating lipid droplet morphology and favoring microvesicular steatosis [31]. Moreover, PCTP, upon complexing with PC, binds and enhances the enzymatic activity of the thioesterase superfamily member 2 (Them2), a mitochondria-associated long-chain acyl-CoA thioesterase. Them2 is also highly expressed in the liver and plays a key role in the rerouting of fatty acids from β-oxidation to glycerolipid biosynthesis [32]. Therefore, induction of *PCTP* by VDR could favor Them2 activity and hence the redirection of fatty acids toward TG synthesis.

The synthesis of TG requires precursors (e.g., fatty acids, glycerol, etc.) that could come from endogenous metabolism or from exogenous sources. The activation of VDR in hepatocytes and HepG2 cells upregulated two important transporters one for fatty acids (*FATP2*) and other for glycerol (*AQP3*).

FATP2 is a member of the FATP family that is predominantly expressed in liver and kidney. Liver-specific *FATP2* knockdown had a significant effect on hepatocyte fatty acid uptake, which was reduced by 40%. Moreover, when these mice were on the HFD, loss of *FATP2* resulted in decreases of liver TGs and intracellular lipid droplets [33].

AQP3 acts as a glycerol channel at physiological pH [34]. The highest expression levels of human *AQP3* were observed in colon, small intestine, kidney and liver [21]. However, liver *AQP3* expression is null in rats [21] and very low in mice [19]. Functional studies of aquaglyceroporins are scarce in humans, and mutations in the genes encoding *AQP3*, *AQP7*, and *AQP9* show divergent phenotypes to those observed in deficient mice [35]. Therefore, the potential role of AQP3 in human hepatocyte lipid metabolism remains to be investigated. We postulated that increased glycerol transport by AQP3 could be another important component in the promotion of TG synthesis in human hepatocytes.

The coordinated upregulation of *FATP2*, *AQP3*, *AGPAT2*, *MOGAT1*, *LPGAT1*, and *DGAT1/2* supports that activated VDR triggers the synthesis of TG in human hepatocytes. This is in agreement with our metabolomic analysis by UPLC-MS. that showed increases in the levels of TG species both at 8 and 24h after VitD. On the contrary, TG precursors such as PAs, MGs, and DGs were found decreased, which is compatible with an induction of enzymes downstream of these precursors.

TG and phospholipids have PA as a common precursor, which is either converted into cytidyldiphosphate diacylglycerol (CDP-DAG) to promote new phospholipid synthesis or is dephosphorylated to produce DG [36]. CDS1 is a key enzyme in this branching point where they convert PA into CDP-DAG for phosphatidylinositol (PI) and phsophatidylglycerol (PG) synthesis. Knocking down *CDS1* increased the amount of PA, triggered the formation of giant lipid droplets and reduce the amount of PI and PG [37]. We observed that activation of VDR in human hepatocytes upregulated *CDS1*. This could redirect some of the PA pool towards CDP-DAG, PIs, and PGs. Our metabolomic analysis, however, confirmed the reduction of PA species, but it did not confirm increases of CDP-DAG, PIs, or PGs. Our results are in agreement with Lykidis et al. [38] showing that CDS1 does not determine the cellular levels of CDP-DAG or PI.

Our metabolomic analysis detected multiple alterations in diacyl phospholipid levels upon VitD. Some PC species augmented whereas other decreased. Regarding PE, results were similar but statistically significant differences were only observed in PE species decreased after VitD. The increase of some PCs could be associated with the upregulation of *SLC6A12* (betaine transporter) and *MAT1A* (S-adenosyl methionine (SAMe) synthesis). These two genes could contribute to an important hepatocyte-specific pathway for PC synthesis that consists in three sequential methylations of PE, catalyzed by PE-N-methyltransferase (PEMT), using SAMe as the methyl donor [39]. The upregulation of SLC6A12 (predominantly expressed in the sinusoidal hepatocyte membrane [40]) should promote the uptake of betaine [41], and betaine has the capacity to elevate hepatic SAMe through the enzyme betaine homocysteine methyltransferase (BHMT) [42], followed by MAT1A, which is also induced by VitD in VDR-hepatocytes. Newly synthesized SAMe can be used to generate PC from PE. Intriguingly, high SAMe levels also promote hepatic TG accumulation, because to maintain a normal membrane PC/PE ratio when SAMe levels are high, the liver stimulates PC secretion via VLDL and increases PC degradation by phospholipase D or C, leading to increased DG and TG production. Thus, excess SAMe levels stimulate both PC synthesis and catabolism, thereby contributing to the development of hepatic TG accumulation [43]. This possibility is supported by studies showing that PC is an unexpected source of TG in the liver through a PC-phospholipase C activity [44].

The hypothesis of increased catabolism of PC is also supported by our finding that the levels of several LPCs were elevated at 8h after VitD in VDR-hepatocytes. LPC is an intermediate in the metabolism of PC, during PC turnover, and is one of the major lipid components of low-density lipoproteins [45,46]. The conversion of LPC from PC is mainly catalyzed by lecithin–cholesterol acyltransferase in blood and phospholipases A1/2 in tissues [46,47]. In turn, LPC can be converted into LPA by lysophospholipase D/Autotaxin [46]. We did not find alterations in the expression of genes involved in LPC metabolism upon VDR activation, and therefore, we cannot suggest a potential mechanism. Nevertheless, we also observed differences in LPCs between non-stimulated VDR hepatocytes and mock transfected hepatocytes, which could suggest a negative effect of non-activated VDR on LPC levels, which would be reverted back upon VDR activation. Therefore, the regulation of LPC levels by VDR and VitD seems to differ from the regulation observed on other lipids.

The increased levels of LPC after VitD could have several consequences. LPC activates multiple signaling pathways that are involved in oxidative stress and inflammatory responses [46]. Moreover, LPC accumulation can promote TG synthesis: systemic supplementation of LPC prior to an oral lipid load decreased hepatic fatty acid oxidation and stimulated TG production [48], and LPC induced TG and phospholipid synthesis, and increased apo B secretion in HepG2 cells [49].

Another intriguing observation is that the intracellular levels of PE plasmalogens (but not PC plasmalogens) decreased in human hepatocytes after 24 h of VDR activation. Plasmalogens, are a subclass of phospholipids in which the hydrocarbon chain at the sn-1 position is attached by an ether bond, instead of an ester bond. Moreover, at the sn-2 position there is a preference for polyunsaturated fatty acids [50,51]. Plasmalogens have unique structural characteristics, which modulate membrane fluidity and membrane fusion. In addition, they are involved in a variety of biological functions, including cell differentiation, cell signaling and oxidative stress [50,51]. Similarly to LPCs, we have not observed alterations in genes for PE ether synthesis, which suggest that the significant changes observed in these lipid classes may be secondary to changes in precursors such as glycerol-3-phosphate or in other central lipids such as PA, DG, or TG. Indeed, the accumulation of TG could be responsible for lower plasmalogens as it has been shown that patients with NAFLD have lower total plasma plasmalogens [52]. Moreover, the plasmalogen/phospholipid ratio of any serum lipoprotein was significantly lower in patients with nonalcoholic steatohepatitis [53]. Plasmalogens are mainly synthesized in liver peroxisomes and secreted into the circulation as part of lipoproteins. The vinyl-ether double bond of plasmalogen is highly sensitive to oxidation, which allow them to act as antioxidants [53]. Therefore, the low level of plasmalogens may also be caused by oxidative stress and peroxisomal dysfunction.

Regarding the clinical translation of our findings we can speculate that high VDR expression and signaling in hepatocytes, in the settings of NAFLD, may contribute to TG accumulation. However, VDR activation in non-parenchymal liver cells like Kupffer or hepatic stellate cells could exert anti-inflammatory and anti-fibrotic effects [54], and consequently prevent NAFLD progression to nonalcoholic steatohepatitis and fibrosis. Thus, the interindividual variability in VDR expression levels in hepatocytes and in non-parenchymal liver cells may determine the outcome of VitD signaling in the liver.

## 5. Conclusions

**Figure 9:**
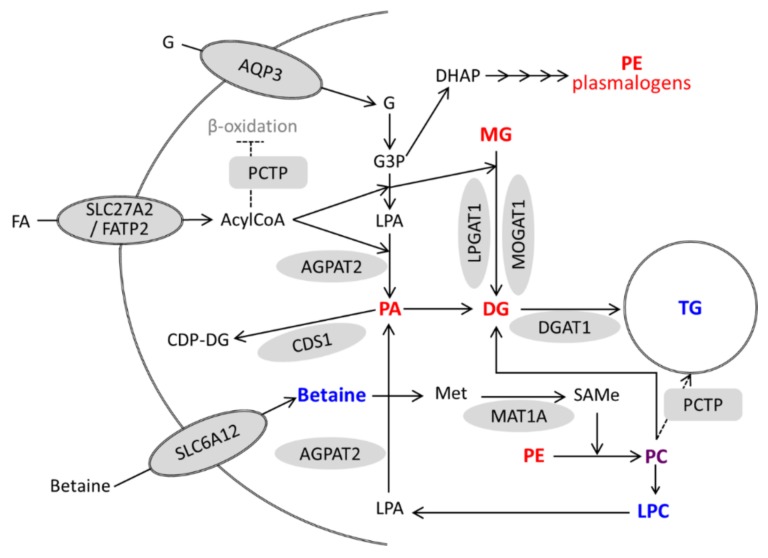
A model for the regulation of lipid metabolism in hepatocytes by activated VDR. Genes upregulated by VitD/VDR are represented by grey forms. Metabolites with increased and decreased levels after VitD are in blue and red, respectively. Phosphatidylcholine (PC) is represented in purple because some species increased and others decreased. G, glycerol; G3P, glycerol-3-phosphate; DHAP, dihydroxyacetone phosphate; FA, fatty acid; LPA, lyso-phosphatidic acid; and Met, methionine.

Activation of VDR in hepatocytes (either by VitD or LCA) triggers a coordinated induction of genes involved in TG synthesis which is mirrored by increases in TG species and decreases in TG precursors. Moreover, activated VDR also induces several genes involved in phospholipid metabolism and remodeling (*PCTP*, *CDS1*, *SLC6A12*, and *MAT1A*), and causes significant alterations in PC species as well as increases in LPCs and decreases in PEs and PE plasmalogens. These alterations in phospholipids could also be involved in (or be a consequence of) TG production and accumulation (Figure 9).

## Figures and Tables

**Table 1:** Gene ontology (GO) terms related to lipid metabolism after GO enrichment analysis of the VitD altered genes in Ad-VDR HepG2 cells.

	GO Term	Category Level	Set Size	Candidates Contained	*p*-Value	q-Value
GO:0033993	response to lipid	BP 4	919	37 (4.0%)	0.000	0.000
GO:0008202	steroid metabolic process	BP 4	315	17 (5.4%)	0.000	0.000
GO:0045444	fat cell differentiation	BP 4	213	11 (5.2%)	0.000	0.003
GO:0008610	lipid biosynthetic process	BP 4	713	23 (3.2%)	0.000	0.003
GO:0030258	lipid modification	BP 4	299	13 (4.4%)	0.001	0.004
GO:0016125	sterol metabolic process	BP 4	158	9 (5.7%)	0.001	0.004
GO:1901654	response to ketone	BP 4	193	9 (4.7%)	0.002	0.012
GO:0006775	fat-soluble vitamin metabolic process	BP 4	42	4 (9.5%)	0.003	0.016
GO:0046486	glycerolipid metabolic process	BP 4	466	15 (3.2%)	0.004	0.018
GO:1905952	regulation of lipid localization	BP 4	136	7 (5.1%)	0.004	0.019
GO:0019216	regulation of lipid metabolic process	BP 5	387	21 (5.4%)	0.000	0.000
GO:0071396	cellular response to lipid	BP 5	608	24 (4.0%)	0.000	0.000
GO:0045598	regulation of fat cell differentiation	BP 5	121	9 (7.4%)	0.000	0.002
GO:0033189	response to vitamin A	BP 5	18	4 (22.2%)	0.000	0.002
GO:0006869	lipid transport	BP 5	342	14 (4.1%)	0.001	0.007
GO:0032368	regulation of lipid transport	BP 5	106	7 (6.6%)	0.001	0.010
GO:0060191	regulation of lipase activity	BP 5	95	6 (6.3%)	0.003	0.020
GO:0050873	brown fat cell differentiation	BP 5	41	4 (9.8%)	0.003	0.020
GO:0002933	lipid hydroxylation	BP 5	7	2 (28.6%)	0.004	0.026
GO:0045834	positive regulation of lipid metabolic process	BP 5	138	7 (5.1%)	0.004	0.026
GO:0008203	cholesterol metabolic process	BP 5	140	7 (5.0%)	0.005	0.028

## Supplementary Materials

The following are available online at https://www.mdpi.com/2218-273X/10/3/493/s1. Figure S1: Response of CYP24A1 to VDR activation in human hepatic cells; Figure S2: Response of novel VDR-target genes to LCA; Figure S3: PCA score plots of Ad-VDR human hepatocytes after incubation with VitD or vehicle for 8 or 24 h; Figure S4: Relative intracellular levs of PCs and PEs in cultured human hepatocytes exposed to VitD; Figure S5: Relative intracellular levs of betaine and phosphatidic acids (PAs) in cultured human hepatocytes exposed to VitD; Table S1: Primers for RT-qPCR in human hepatocytes and mouse livers; Table S2: Official names and roles of the novel genes induced by activated VDR in human hepatocytes.

## Abbreviations

VDR	vitamin D receptor
VitD	vitamin D
LCA	lithocholic acid
Ad	adenovirus
HFD	high-fat diet
FDR	false discovery rate
PCA	principal component analysis
MG	monoacylglycerol
DG	diacylglycerol
TG	triglyceride
PA	phosphatidic acid
PC	phosphatidylcholine
PE	phosphatidylethanolamine
LPC	lysophosphatidylcholine
LPE	lysophosphatidylethanolamine
NAFLD	nonalcoholic fatty liver disease
