# Supplementary material for: The Vitamin D Receptor Regulates Glycerolipid and Phospholipid Metabolism in Human Hepatocytes
Source: Biomolecules. 2020 Mar 24;10(3):493. doi: 10.3390/biom10030493 (PMC7175212; doi:10.3390/biom10030493)
Supplement: Supplementary file 1 [file biomolecules-10-00493-s001.pdf]

Supplemental information to:

The Vitamin D Receptor Regulates Glycerolipid and Phospholipid Metabolism in Human Hepatocytes

Teresa Martínez-Sena[1], Polina Soluyanova [1], Carla Guzmán [1], José Manuel Valdivielso [2], José Vicente Castell [1,3,4] and Ramiro Jover [1,3,4] *

[1] Experimental Hepatology Unit, IIS Hospital La Fe, Valencia, E-46026, Spain; teresa_martinez@iislafe.es (T.M.-S.), Polina.Soluyanova@hotmail.com (P.S.), carla_teclab@yahoo.es (C.G.), jose.castell@uv.es (J.V.C.)

[2] Vascular and Renal Translational Research Group, Experimental Medicine Department, IRBLleida, Lleida, E-25196, Spain; josemanuel.valdivielso@udl.cat

[3] Departamento de Bioquímica y Biología Molecular, Facultad de Medicina, Universidad de Valencia, Valencia, E-46010, Spain

[4] Centro de Investigación Biomédica en Red de Enfermedades Hepáticas y Digestivas (CIBERehd), ISCIII, Madrid, E-28029, Spain.

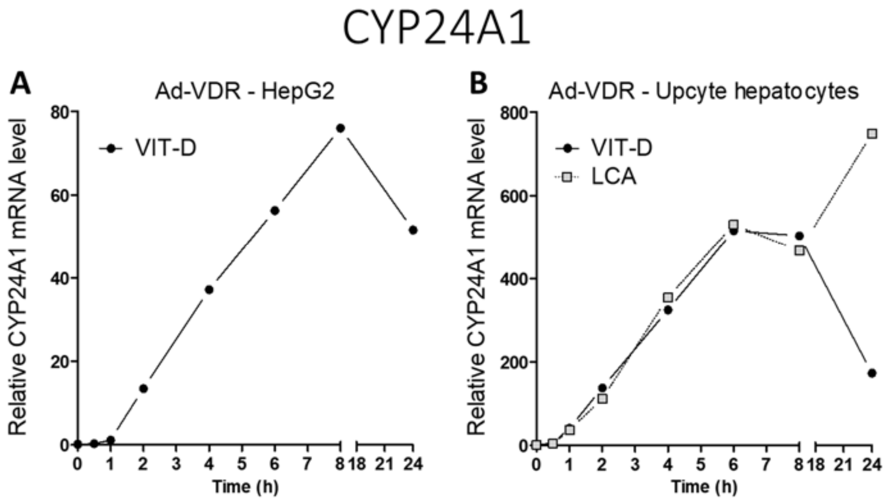

Supplemental Figure S1: ***Response of CYP24A1 to VDR activation in human hepatic cells***. HepG2 cells (**A**) and upcyte hepatocytes (**B**) were infected with Ad-VDR and, 48 h later, 10 nM calcitriol (VIT-D) or 100 µM LCA was incorporated. Total RNA was purified at different time-points and the mRNA level of the VDR-target gene *CYP24A1* (25-Hydroxyvitamin D-24-hydroxylase) was determined by RT-qPCR. Data represent the *CYP24A1* mRNA level normalized with the housekeeping *PBGD* & *RPLP0* mRNAs, and are expressed as fold-change relative to parallel, control adenovirus-infected cells.

Supplemental UPLC-MS methods:

Acetonitrile (CH_3CN, LCMS grade) was obtained from Scharlau (Barcelona, Spain), Chloroform ($CHCl_3$, ≥99%) from Sigma-Aldrich and 2-Propanol (IPA, LCMS grade) from Fisher Scientific (Loughborough, UK). Formic acid (HCOOH, ≥95%) and ammonium formate (NH_4HCO_2, ≥99%) were obtained from Sigma-Aldrich (Madrid, Spain). Ultra-pure water was generated employing a Milli-Q Integral Water Purification System from Merck Millipore (Darmstadt, Germany). Reserpine was purchased from Sigma-Aldrich (Madrid, Spain) and Phenylalanine-D_5, Tryptophan-D_5 and Caffeine-D_9 were purchased from CDN Isotopes (Sainte Foy La Grande, France).

In the chromatographic analysis, autosampler and column temperatures were set to 4°C and 55°C, respectively. Injection volume was 4 µl and the flow rate was 400 µl/min. The electrospray ionization parameters were the following: gas T, 200°C; drying gas, 14 L/min; nebulizer, 37 psig; sheath gas T, 350°C; sheath gas flow, 11 L/min. Automatic MS spectra calibration during the analysis was carried out by introducing m/z references 149.02332 (phthalic anhydride), 121.050873 (purine) and 922.009798 (HP-0921) into the source via sprayer valve. QCs were repeatedly analyzed at the beginning of the batch analysis using the automatic MSMS acquisition mode with the following inclusion m/z precursor ranges: 70-200, 200-350, 350-500, 500-650, 650-800, 800-950, 950-1100 and 1100-1200. MSMS analysis was carried out at 3 Hz in centroid mode in extended dynamic range mode (2 GHz) with 25V as collision energy enabling the acquisition of 3738 MSMS and 5330 MSMS spectra from the metabolomic and lipidomic methods, respectively. MS isotopic profiles, MSMS spectra and an in-house library of compounds were used to support the putative identification of metabolites using the HMDB and LipidBlast reference databases and in-house written and LipiDex software. Seven QC replicates were injected at the beginning of each batch for system conditioning and every 6 samples to monitor and correct changes in the instrument response. Samples were randomly analyzed within each batch.

The parameters used for the alignment and deconvolution of the signals were the same for the metabolomic and the lipidomic analyses: centWave algorithm, precision of the mass: 15 ppm, width of the peak: (10,40), snthresh: 10, prefilter: (2,2000), mexican hat wavelet was used for peak detection.

Retention time correction was carried out with the nearest method using rtCheck = 6s. Within batch effect correction was carried out using the Quality Control - Support Vector Regression (QC-SVRC) approach using the following intervals for the optimization of the epsilon: [2.5:0.5:7.5], gamma: 10[0:5] and C=50 hyperparameters, and leave-one-out cross validation for SVR model selection. UPLC-MS features with RT in the [0.5,11.5] and [0.5,13.0] min ranges in the metabolomic and lipidomic analysis, respectively, were retained for further analysis. Data cleaning left 571 and 738 UPLC-MS features and putative identification lead to the annotation of 272 and 218 signals in the metabolomic and lipidomic data sets, respectively. Among them, phospholipids (primarily phosphatidylcholines (PC) and phosphatidylethanolamines (PE)), lyso-phospholipids (primarily LPC and LPE), ether phospholipids (plasmalogens), monoacylglycerols (MG), diacylglycerols (DG) and TG were the most common lipids identified. Low-level data fusion was carried out by concatenation of the two sources. The pre-processing and analysis of the data was developed in MATLAB 2017b (Mathworks Inc., Natick, USA) and the PLSToolbox 8.3 (Eigenvector Res.Inc., Wenatchee, USA). Principal component analysis (PCA) was carried out using autoscaling as preprocessing.

Supplemental Table S1: Primers for RT-qPCR in human hepatocytes and mouse livers.

**HUMAN GENE**	**ACCESSION №**	**FORWARD**	**Forw. 5'nt**	**REVERSE**	**Rev. 5'nt**
PBGD	NM_000190	CGGAAGAAAACAGCCCAAAGA	185	TGAAGCCAGGAGGAAGCACAGT	478
RPLP0	NM_001002	ACAACCCAGCTCTGGAGAAA	289	TGCCCCTGGAGATTTTAGTG	528
MOGAT1	NM_058165	CTTTGCCCCTGTTTCATGCC	901	AGTCTCGTGCTCTGGAATGC	1121
DGAT1	NM_012079	AAGAGGAGGTGCGGGACG	299	ATGCCACGGTAGTTGCTGAA	479
AGPAT2	NM_006412	GGGAGAACCTCAAAGTGTGGA	585	GTACACCACGGGGACGATG	712
LPGAT1	NM_001320808	GATGTACCCCTGGAGACTGAT	1787	CACAAGTTGCTGAGGGTCAT	1944
CDS1	NM_001263	TGGATTCATTGCTGCCTATGTG	1145	TGCTGTGGATCTGGAAAGGGTA	1344
SLC27A2 / FATP2	NM_003645	TGCCCTTTTACCACAGTGCTGC	1023	AGCCATTTCCCAGTGCCAGTCT	1253
SLC6A12	NM_003044	GGCGGACCGTTTCTATGACA	2350	AGGAGGGTGATGACGACGAA	2595
AQP3	NM_004925	CTGAACCCTGCCGTGACCT	307	AAGCTGGTTGTCGGCGAAGT	468
PCTP	NM_021213	GACTGCGGAAGGATGGAGC	42	TGCCAGTAGAGTTGGTGAGC	260
MAT1A	NM_000429	CTGCGGCCTGACTCTAAGAC	761	GGTAGACGGTGTCTTCGTCC	962
**MOUSE GENE**	**ACCESSION №**	**FORWARD**	**Forw. 5'nt**	**REVERSE**	**Rev. 5'nt**
Gapdh	NM_001289726	AGGGCTCATGACCACAGTCCAT	602	GCCAGTGAGCTTCCCGTTCAG	776
Rplp0	NM_007475	GGGTACAAGCGCGTCCTG	851	TTGGCGGGATTAGTCGAAGAG	1084
Mogat1	NM_026713	AATGGGAGTAGCCTTGCCAC	885	CAATCTGCTCTGAGGTCGGG	1030
Dgat1	NM_010046	CCTGAATTGGTGTGTGGTGA	504	AGGGGTCCTTCAGAAACAGAG	625
Agpat2	NM_026212	CCCGTGGTGTACTCGTCTT	808	CATTGGTAGGGACAGCATCCA	910
Lpgat1	NM_001134829	GTAGCACGGCAGGAAAATGG	906	ACTGTTGGTTTCCTGTACCCC	1066
Cds1	NM_173370	AGCAAACAACCACCGTGAGA	3432	TTCCTGGTTGCCACTCATCC	3532
Slc27a2 / Fatp2	NM_011978	AGTGTCGGCGGAACCCA	731	TGTCCCATACCATAGGCGATGA	869
Slc6a12	NM_133661	TGGTCACAGAGGGCGGGAT	1633	AAACGGTCGGCCCCATACAC	1759
Aqp3	NM_016689	GGCGCTGGGATTGTTTTTGG	417	CACACACAATAAGGGCGGCT	597
Pctp	NM_008796	ACCCTTTCCCGCTGTCCAAC	366	TCTCGATCGCCAGGCTCTGT	539
Mat1a	NM_133653	AAGTGGGCGGTTTGTTATTG	1213	CAGCCTTTACCAGGGACTTG	1400

Supplemental Table S2: Official names and roles of the novel genes induced by activated VDR in human hepatocytes.

**GENE**	**Name**	**Role / Pathway**
MOGAT1	monoacylglycerol O-acyltransferase 1	Glycerolipid metabolism.
DGAT1	diacylglycerol O-acyltransferase 1	Glycerolipid metabolism.<br>Retinol metabolism.
AGPAT2	1-acylglycerol-3-phosphate O-acyltransferase 2	Glycerolipid metabolism.<br>Glycerophospholipid metabolism.
LPGAT1	lysophosphatidylglycerol acyltransferase 1	Glycerophospholipid metabolism.
CDS1	CDP-diacylglycerol synthase 1	Glycerophospholipid metabolism.
SLC27A2 / FATP2	solute carrier family 27 member 2	Fatty acid transporter & long/very long chain acyl-CoA synthetase.
SLC6A12	solute carrier family 6 member 12	Betaine / GABA transporter.
AQP3	aquaporin-3	Aquaglyceroporin or glycerol-uptake facilitator.
PCTP	phosphatidylcholine transfer protein	Phospholipid binding protein.
MAT1A	methionine adenosyltransferase 1A	S-adenosylmethionine synthetase.

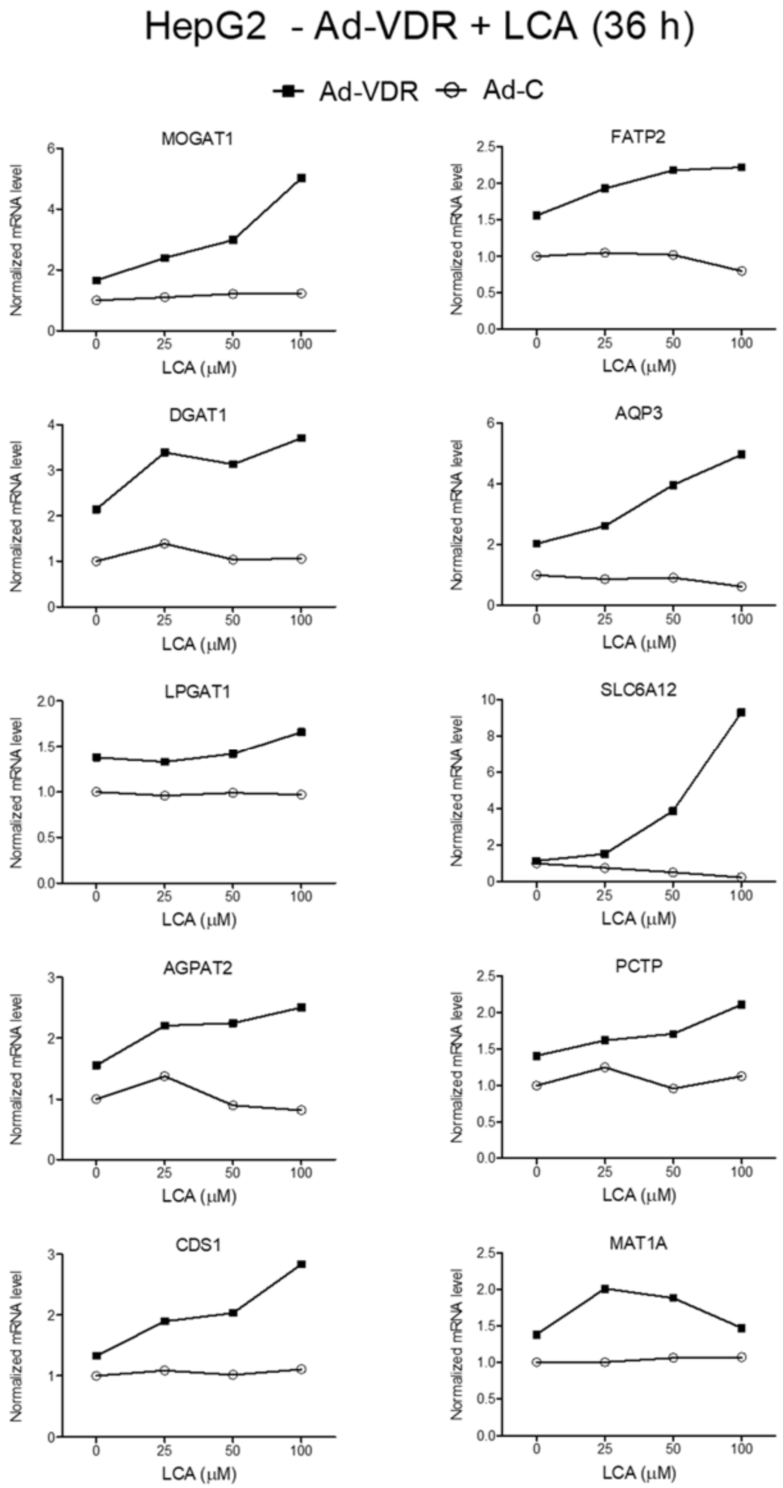

Supplemental Figure S2: ***Response of novel VDR-target genes to LCA***. HepG2 cells were infected with Ad-C or Ad-VDR and, 48 h later, LCA was incorporated for 36 h more. Total RNA was purified at different time-points and the mRNA level of novel VDR-regulated genes was determined by RT-qPCR. Data represent mRNA levels, after normalization with the housekeeping genes *PBGD* & *RPLP0* mRNAs, and are expressed as fold-change relative to Ad-C cells without LCA.

A

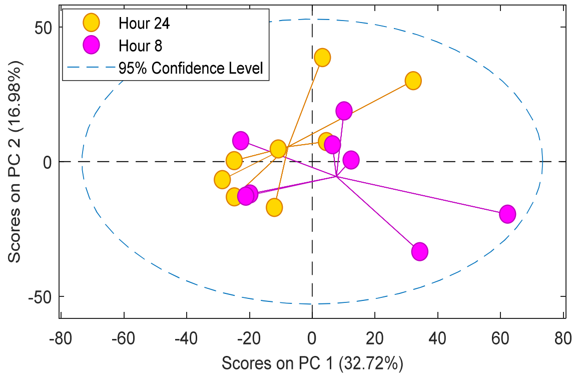

B

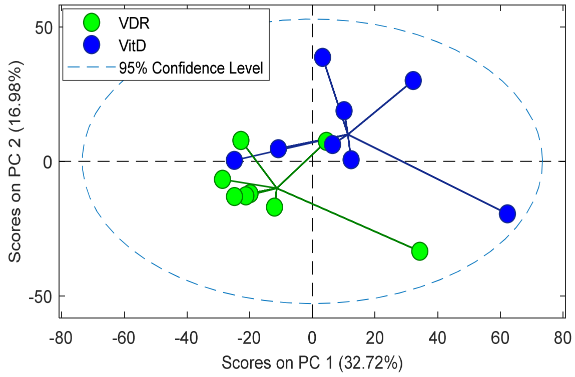

Supplemental Figure S3*. PCA score plots of Ad-VDR human hepatocytes after incubation with VitD or vehicle for 8 or 24h.* **(A)** Variance caused by time. **(B)** Variance caused by VitD.

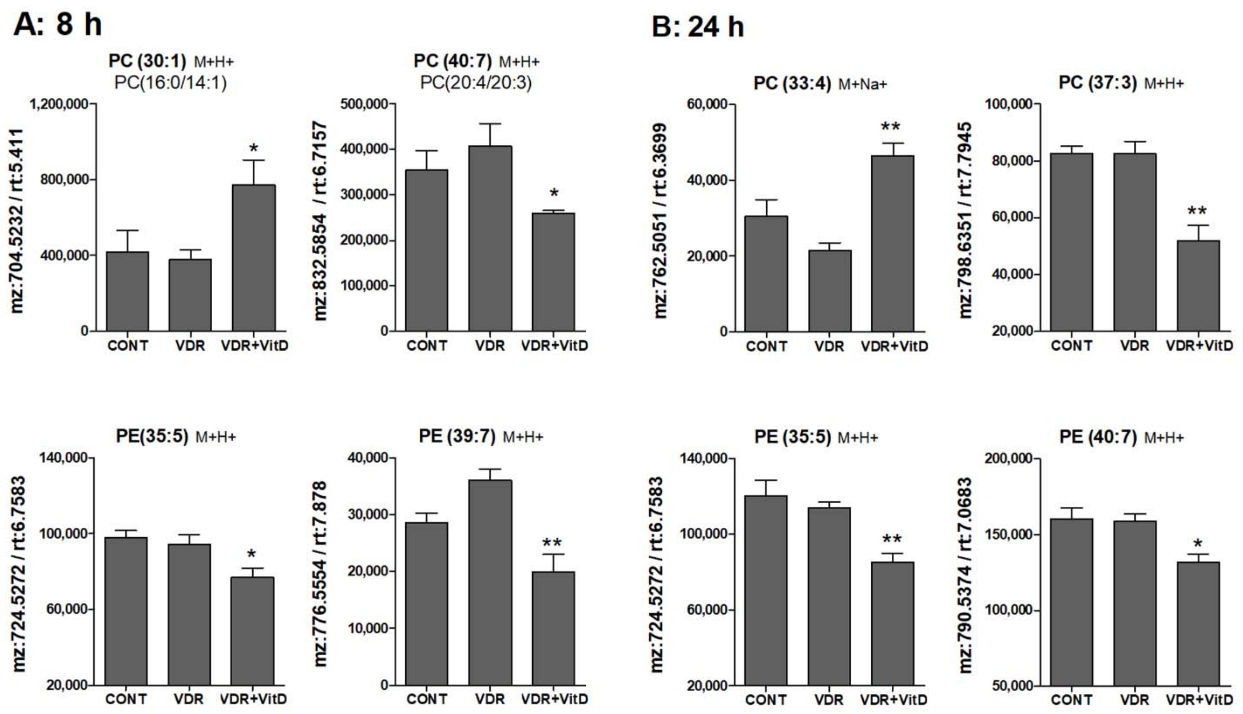

Supplemental Figure S4. ***Relative intracellular levs of PCs and PEs in cultured human hepatocytes exposed to VitD.*** Upcyte hepatocytes were infected with Ad-C or Ad-VDR for 48 h. Then, 10 nM VitD (VDR+VitD, n=4) or vehicle (CONT, n=3; VDR, n=4) were added for 8 or 24 h. Cells were washed, and intracellular metabolites extracted and analyzed by UPLC/MS as described in Materials and Methods. *p<0.05 and **p<0.01 VDR+VitD vs VDR.

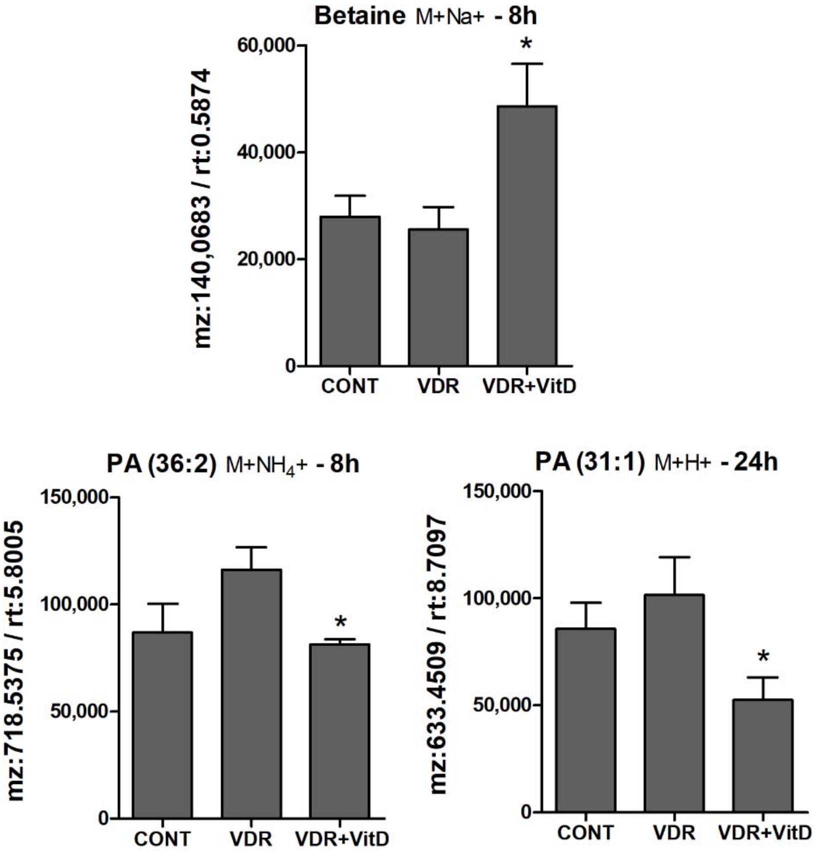

Supplemental Figure S5. ***Relative intracellular levs of betaine and phosphatidic acids (PAs) in cultured human hepatocytes exposed to VitD.*** Upcyte hepatocytes were infected with Ad-C or Ad-VDR for 48 h. Then, 10 nM VitD (VDR+VitD, n=4) or vehicle (CONT, n=3; VDR, n=4) were added for 8 or 24 h. Cells were washed, and intracellular metabolites extracted and analyzed by UPLC/MS as described in Materials and Methods. *p<0.05 VDR+VitD vs VDR.
